# Callus Culture of *Scorzonera radiata* as a New, Highly Productive and Stable Source of Caffeoylquinic Acids

**DOI:** 10.3390/molecules27227989

**Published:** 2022-11-17

**Authors:** Olga V. Grishchenko, Valeria P. Grigorchuk, Galina K. Tchernoded, Olga G. Koren, Victor P. Bulgakov

**Affiliations:** Federal Scientific Center of the East Asia Terrestrial Biodiversity, Far Eastern Branch of Russian Academy of Sciences, 690022 Vladivostok, Russia

**Keywords:** *Scorzonera radiata*, callus line, caffeoylquinic acids, secondary metabolites, caffeoylquinic acids

## Abstract

During our ongoing efforts to investigate biotechnological sources of caffeoylquinic acid (CQA) metabolites, we discovered the plant *Scorzonera radiata* Fisch. (Asteraceae), which is able to produce callus cultures with high yield and extremely high stability. An actively growing callus line, designated as Sr-L1, retained the ability to produce 11 CQAs during long-term cultivation (more than 20 years). A total of 29 polyphenolic compounds were identified in the leaves and Sr-L1 callus culture of *S. radiata*, including CQAs, lignol derivatives, flavonoids, and dihydrostilbenes. The composition of CQAs in the Sr-L1 culture was identical to that in the *S. radiata* leaves. Sr-L1 calli did not produce flavonoids and dihydrostilbenes, but produced lignol derivatives, which were absent in leaves. The HPLC-UV-HRMS determination showed the presence of monoacyl derivatives of CQAs such as 5-CQA, 4-CQA, *cis*-5-CQA, and 5-*O*-*p*-coumaroylquinic acid in the Sr-L1 culture. Among diacyl derivatives, 3,4-diCQA, 3,5-diCQA, *cis*-3,5-diCQA, 4,5-diCQA, 3-*O*-*p*-coumaroyl-5-*O*-CQA, and 3-*O*-caffeoyl-5-*O*-p-coumaroylquinic acid were found. The content of 5-CQA reached 7.54 mg/g dry weight and the content of 3,5-diCQA was as high as 18.52 mg/g dry weight. 3,5-diCQA has been reported to be of high nutritional and pharmacological value, as it alleviates inflammatory pain, reverses memory impairment by preventing neuronal apoptosis, and counteracts excessive adipose tissue expansion, serving as an attractive treatment option for obesity. The high content of 3,5-diCQA and the exceptional stability of biosynthesis make callus cultures of *S. radiata* a promising source for the development of drugs and nutraceuticals.

## 1. Introduction

The genus *Scorzonera* L. (Asteraceae) is represented by approximately 180–190 species widely distributing in the temperate and subtropical regions of Europe, Asia, and North Africa [1,2]. Many *Scorzonera* species have long been used in folk medicine of European countries, Mongolia, and China, and also employed as food plants in these regions [3,4,5,6]. Caffeoylquinic acids (CQAs) are most typical for plants of the Asteraceae family; in particular, high content of CQAs is known for artichoke (*Cynara scolymus*), black salsify (*Scorzonera hispanica* L.), purple coneflower (*Echinacea purpurea* L. Moench), common yarrow (*Achillea millefolium* L.), milk thistle (*Silybum marianum* L. Gaertner), coltsfoot (*Tussilago farfara* L.), tansy (*Tanacetum vulgare* L.), and chamomile (*Matricaria chamomilla* L.) [7]. A recent review by Lendzion et al. [8] summarized data on phytochemistry and bioactivity of 37 species and subspecies of the genus *Scorzonera*. CQAs were found in 25 examined plant taxa. In most *Scorzonera* species, only chlorogenic acid (CGA) was identified (in 19 species). Among diacyl derivatives, 3,5-diCQA was found most frequently in *Scorzonera* species (11 species). The greatest diversity of CQAs was found in *S. radiata* and *S. hispanica* [8]. Structural diagnostic hierarchical keys for CQA were developed by Clifford et al. [9,10,11,12,13] using the name “chlorogenic acid” in accordance with the early IUPAC nomenclature rules [14]. Many authors use the more recent names “neochlorogenic acid” and “chlorogenic acid” for 5-*O*-CQA and 3-*O*-CQA according to the PubChem database and chemical abstract (CAS) [15]. The nomenclature differences are explained in the comprehensive review by Magaña et al. [16] for the name of chlorogenic acid (3-*O*-CQA). We also used new nomenclature, but when discussing the results and comparing our and literature data, we took into account these disagreements.

*S. austriaca*, traditionally used in Chinese and Tibetan herbal medicine due to its antipyretic and anti-inflammatory action [17,18,19], was selected by the National Committee of China as a drug candidate for the treatment of hepatitis B because the total flavonoid fraction isolated from *S. austriaca* has been shown to have hepatoprotective and inhibitory effects on the hepatitis B virus [19]. Sezer et al. [20] investigated the neuroprotective potential of 27 taxa of the genus *Scorzonera* to find new sources of biologically active substances for the treatment of neurodegenerative diseases. They concluded that at least one of the species, *S. pisidica*, could be recommended for further investigation of its neuroprotective action [20]. Thus, the pharmacological effects and experience of using *Scorzonera* plants make them good candidates for drug discovery as well as nutritional supplement development. Phytochemical studies of *Scorzonera* species have revealed a number of secondary metabolites, such as stilbene derivatives, sesquiterpene lactones, lignans, phenolic acids, flavonoids, dihydroisocoumarins, and triterpenes [3,4,8,21,22,23,24,25].

In the present study, plants of *S. radiata* Fisch. were used. This plant is interesting due to the variety of secondary metabolites and many years of experience in traditional medicine. *S. radiata* is widely distributed in Mongolia and China [5] as well as in southeastern Russia [5,26]. The chemical composition of *S. radiata* was studied by a group of researchers [5,26,27]. From the aerial parts of the plant, they isolated dihydrostilbenes, flavonoids, and derivatives of quinic acid including diCQAs, as well as coumarins, simple benzoic acids, and one monoterpene glycoside. They also studied the antioxidant activity of the isolated substances and found high antioxidant activity of two dihydrostilbenes (scorzodihydrostilbenes A and E) and diCQAs [5,26,27]. It is known that the qualitative and quantitative composition of plant metabolites of the same species can vary depending on different factors, including the region of growing [28,29]. Therefore, the development of cell cultures with stable biosynthetic characteristics is a known approach to obtain a renewable source of secondary metabolites. Only one representative of the genus *Scorzonera* (*S. hispanica*) was introduced into the in vitro culture. A suspension culture of transformed *S. hispanica* cells producing a complex of lignans and neolignan glucosides was established [30,31]. Previously, cell cultures of *S. radiata* were not obtained; here we report the establishment of the first callus culture from this plant.

Our goal was to obtain a callus culture of *S. radiata* as a reproducible source of useful metabolites. In this study, we presented the composition of polyphenolic compounds in the *S. radiata* callus line, designated Sr-L1. We showed the exceptionally high stability of CQA biosynthesis in the Sr-L1 callus line. Two valuable compounds, 5-CQA and 3,5-diCQA, were produced by this culture in high yields. In addition, the chemical composition of *S. radiata* plants collected on Sakhalin Island was studied and compared with literature data.

## 2. Results

### 2.1. Callus Line Sr-L1

**Figure 1:**
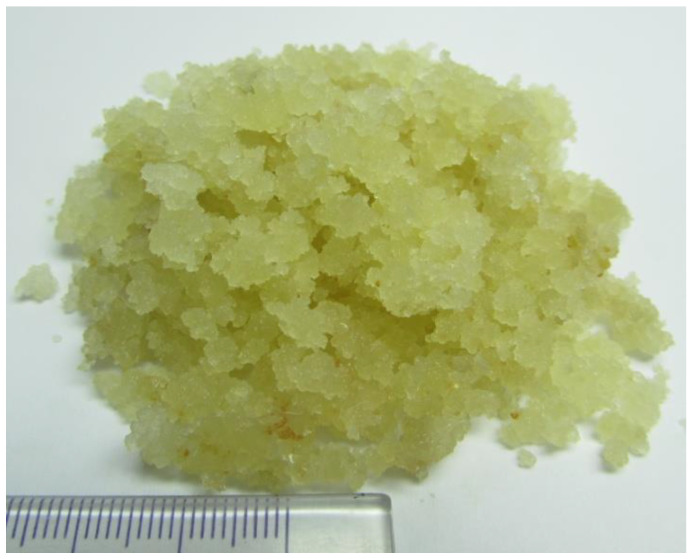
View of the Sr-L1 callus line of *Scorzonera radiata* at the stationary phase of growth, 30 days.

The callus line Sr-L1 of *S. radiata* was selected as the most actively growing culture. The selection was carried out in the usual way, that is, the selection of actively growing cell aggregates during the first years of cultivation. This line has been maintained by subculturing in the Plant Cell Culture Collection of the Federal Scientific Center for Terrestrial Biodiversity of East Asia (Vladivostok, Russia) for 20 years without any additional manipulations and has retained its excellent growth characteristics all this time. Sr-L1 produces a friable, vigorously growing callus of light green-yellow color (Figure 1).

The calli demonstrate the usual growth dynamics with an exponential phase of growth, a linear phase, and a stationary phase ending on day 34–37 by the time of collecting calli for drying. The growth index of the Sr-L1 callus line was 16.5 ± 0.06, with an inoculum mass of 0.2 g. Dry biomass obtained by drying calli at 50 °C to constant weight was 3.15% of fresh biomass.

### 2.2. Analysis of Secondary Metabolites in the Sr-L1 Callus Line and Leaves of S. radiata Plants

**Figure 2:**
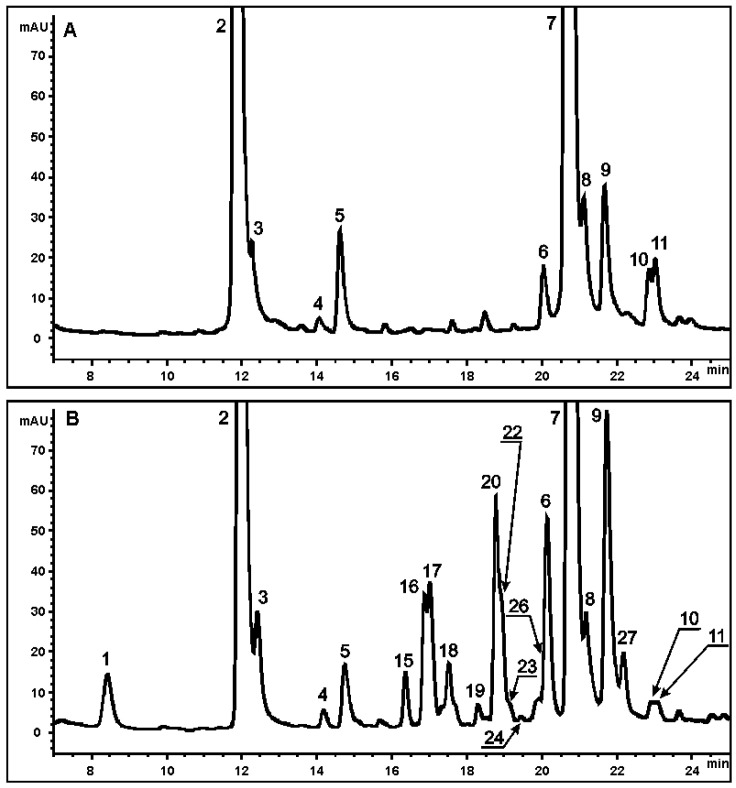
Representative HPLC-UV profiles of *S. radiata* extracts, monitored at 325 nm. (**A**) Methanolic extract of the Sr-L1 callus line; (**B**) methanolic extract of *S. radiata* leaves. Peak numbers correspond to each of the identified components listed in Table 1, Table 2, Table 3 and Table 4.

**Figure 3:**
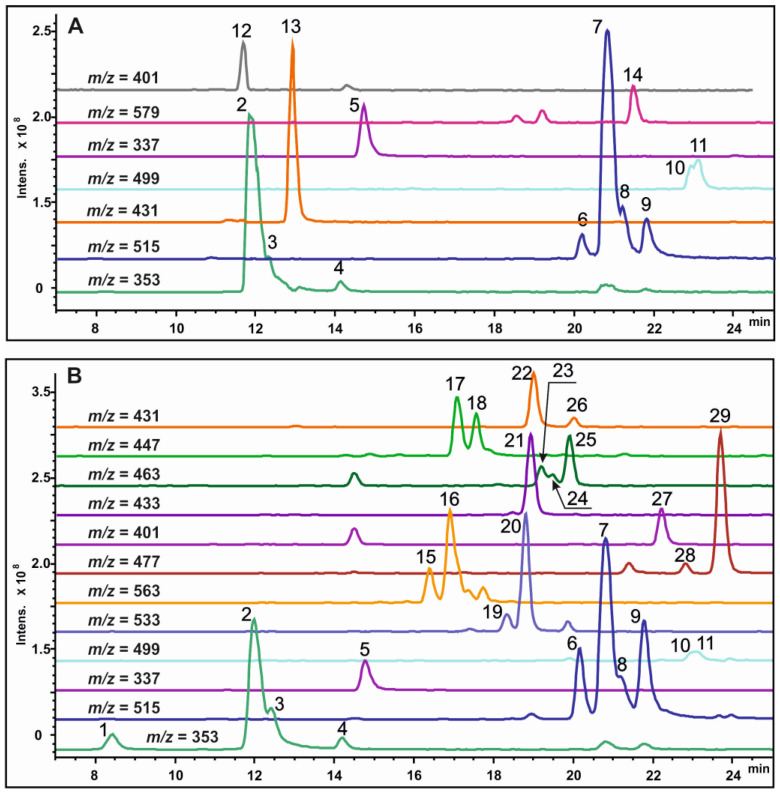
The HPLC-ESI-MS analysis of extracts obtained from the Sr-L1 callus line (**A**) and leaves (**B**) of *S. radiata*. Extracted ion chromatograms of the identified components in negative ion detection mode are presented. The monitored ions correspond to the most abundant deprotonated molecules [M-H]^−^ for **1–11** and **14–29**, and acetylated molecular ions [M+CH_3_COO]^−^ for **12** and **13**. Peak numbers correspond to each of the identified components listed in Table 1, Table 2, Table 3 and Table 4.

HPLC-UV and ESI-MS chromatograms of crude methanol extracts of callus tissues and leaves of *S. radiata* are presented in Figure 2 and Figure 3.

A total of 29 substances were detected in extracts of the Sr-L1 callus line and leaves of *S. radiata*: 14 in the Sr-L1 line and 26 in leaves. Eleven compounds found were common to both callus and leaf extracts. All determined components of the extracts were identified on the base of UV spectra, recorded with a DAD detector, mass spectral data, and chromatographic separation using a reverse phase column. The values of the monoisotopic molecular masses were obtained using the high-resolution mass spectrometry. Based on these mass data, molecular formulas were obtained that are in good agreement with the theoretical data for each found component. A mass error was less than 5 ppm, thus confirming the elemental composition of each component. To detect the characteristic fragmentation patterns of regioisomers, multistage MS analysis was performed, and target fragmentation spectra were obtained in the negative ion mode using an ion trap mass spectrometer. For the qualitative determination of all peaks, their ESI-MS data were compared with those of chemical standards and/or literature data, and each peak was assigned on the basis of the structural diagnostic hierarchical keys previously developed [9,10,11,12,13,32]. All identified metabolites belonged to four classes: CQAs, lignans (presented as lignol derivatives), flavonoids, and stilbenoids (presented as dihydrostilbene derivatives). A total of 11 CQAs were found in the Sr-L1 line and leaves of *S. radiata* (Table 1), 3 lignol derivatives were found in the Sr-L1 line, and 15 flavonoids and dihydrostilbene derivatives were found in the leaves of *S. radiata*. Thus, calli produced CQAs and lignol derivatives, but did not produce flavonoids and dihydrostilbenes. The leaves, in turn, did not contain lignols.

**Table 1:** List of caffeoyl- and coumaroylquinic acid derivatives identified in methanol extracts of Sr-L1 callus culture and *S. radiata* leaves by their retention times and UV and MS data.

Peak No.	*t*_R_ (min)	λ_max_ (nm)	[M-H]^−^ (*m/z* Detected)	[M-H]^−^ (*m/z* Calculated)	Molecular Formula	MS^2^ Fragmentation (Precursor Ions [M-H]^−^) (% Base Peak) (*m/z*)	MS^3^ Fragmentation (% Base Peak) (*m/z*) **	MS^4^ Fragmentation (% Base Peak) (*m/z*) **	Assignment
**1**	8.4	324	353.0888	353.0878	C_16_H_18_O_9_	**191(100)** *, 179(46), 135(6)	173(91), 155(41), 127(82), 111(48), 93(73), 85(100)	-	3-*O*-CQA
**2**	11.9	325	353.0892	353.0878	C_16_H_18_O_9_	**191(100)**, 179(2)	173(100), 127(80), 111(46), 109(48), 93(48), 85(94)	-	5-*O*-CQA
**3**	12.4	325	353.0889	353.0878	C_16_H_18_O_9_	191(38), 179(62), **173(100)**, 135(5)	155(35), 137(18), 111(30), 93(100)	-	4-*O*-CQA
**4**	14.2	320	353.0887	353.0878	C_16_H_18_O_9_	**191(100)**, 179(2)	173(100), 127(75), 111(26), 109(27), 93(42), 85(92)	-	*cis*-5-*O*-CQA
**5**	14.7	315	337.0939	337.0929	C_16_H_18_O_8_	**191(100)**, 163(3)	173(93), 127(100), 111(40), 93(48), 85(88)	-	5-*O*-*p*-coumaroylquinic acid
**6**	20.1	326	515.1213	515.1195	C_25_H_24_O_12_	**353(100)**, 335(9), 299(1), 255(3), 203(5), 179(11), 173(30)	191(50), 179(61), **173(100)**, 135(10)	155(65), 111(23), 93(100)	3,4-*O*-diCQA
**7**	20.7	326	515.1207	515.1195	C_25_H_24_O_12_	**353(100)**, 191(5)	**191(100)**, 179(38), 173(4), 135(6)	173(100), 127(75), 111(63), 85(83)	3,5-*O*-diCQA
**8**	21.1	325	515.1208	515.1195	C_25_H_24_O_12_	**353(100)**, 191(6)	**191(100)**, 179(32), 173(3), 135(5)	173(100), 127(70), 111(55), 85(81)	*cis*-3,5-*O*-diCQA
**9**	21.7	326	515.1206	515.1195	C_25_H_24_O_12_	**353(100)**, 335(3), 317(4), 299(5), 255(3), 203(11), 179(8), 173(10)	191(29), 179(53), **173(100)**, 135(7)	111(60), 93(100)	4,5-*O*-diCQA
**10**	22.9	316	499.1259	499.1246	C_25_H_24_O_11_	353(5), **337(100)**, 335(3), 173(5), 163(12)	173(59), **163(100)**, 119(6)	119(100)	3-*O*-*p*-coumaroyl-5-*O*-CQA
**11**	23.1	317	499.1257	499.1246	C_25_H_24_O_11_	**353(100)**, 337(21), 191(5), 179(5)	**191(100)**, 179(16)	173(100), 127(54), 111(68), 93(42), 85(64)	3-*O*-caffeoyl-5-*O*-*p*-coumaroylquinic acid

* Precursor ions for the subsequent MS^n^ fragmentation are shown in bold. ** The characteristic fragmentation patterns of regioisomers are shown.

### 2.3. Determination and Quantification of CQAs

Among detected compounds, 11 CQAs were identified (Figure 2 and Figure 3). Five monoacyl (peaks **1–5**) and six diacyl (peaks **6–11**) CQAs were identified according to a previously described approach [33] based on comparison of their MS^n^ fragmentation patterns, elution/retention time, and UV absorption spectra with standards and published data [9,10,11,12,13,32]. Chromatographic and spectral characteristics of the studied metabolites are presented in Table 1.

**Figure 4:**
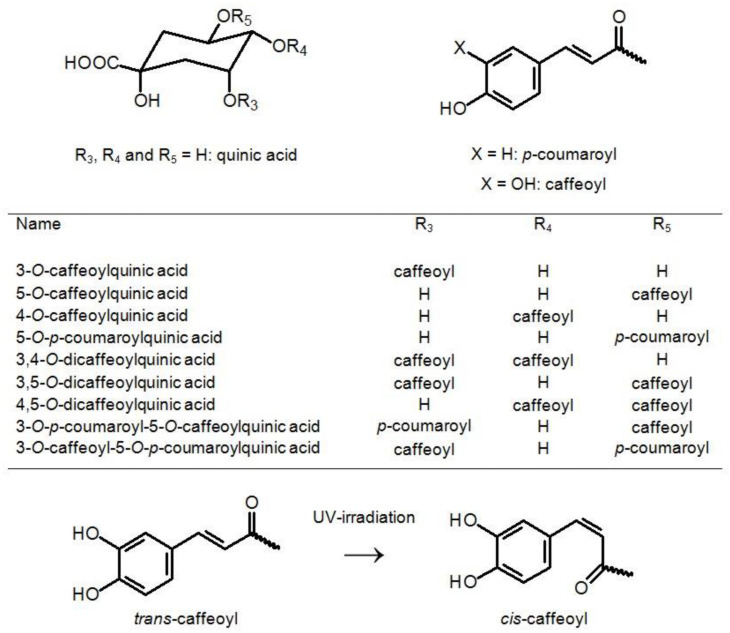
Structures of caffeoylquinic acids found in *S. radiate* plants and calluses. A scheme explaining geometric isomerization of caffeoyl moieties under UV irradiation is presented.

According to the hierarchical keys previously developed [9,10,11,12,13,32] and reference standards, all eleven CQAs were designated as 3-*O*-CQA (**1**), 5-*O*-CQA (**2**), 4-*O*-CQA (**3**), *cis*-5-*O*-CQA (**4**), 5-*O*-*p*-coumaroylquinic acid (**5**), 3,4-*O*-diCQA (**6**), 3,5-*O*-diCQA (**7**), *cis*-3,5-*O*-diCQA (**8**), 4,5-*O*-diCQA (**9**), 3-*O*-*p*-coumaroyl-5-*O*-CQA (**10**), and 3-*O*-caffeoyl-5-*O*-*p*-coumaroylquinic acid (**11**) (Table 1). Compounds **4** and **8,** presented in minor amounts and showing the MS^n^ fragmentation pattern similar to those of compounds **2** and **7**, respectively, were tentatively assigned to the cis isomers of compounds **2** and **7.** Since it is known that chlorogenic acid undergoes trans–cis isomerization under the action of UV light [13], we tested this assumption in experiments with UV irradiation of all samples. After UV irradiation of extracts, the compounds **4** and **8** were detected in chromatograms as peaks with a significant increased intensity, which confirmed the presence of two *cis* isomers in our samples, *cis*-5-*O*-CQA (**4**) and *cis*-3,5-*O*-diCQA (**8**). Figure 4 presents the general structures of CQAs, diCQAs, and *p*-coumaroylquinic acids in *S. radiate* plants and the Sr-L1 callus line.

All identified CQAs were quantified using HPLC with UV detection at a wavelength of 325 nm on the basis of four-point regression curves built with the reference commercial standards. Chlorogenic acid was used for quantitative determination of monoacyl derivatives, and 1,3-*O*-diCQA (cynarin) was used for quantification of diacyl derivatives. The content of CQAs in the *S. radiata* Sr-L1 callus line and leaves is presented in Table 2. It should be noted that these measurements were started in November 2014 during the revision of callus cultures of various plant species from the Sakhalin collection. It was surprising to find that the Sr-L1 callus culture showed a high content of secondary metabolites, since by this time 13 years had passed after establishing the culture. Table 2 presents a summary of quantification data for the period 2014–2022. Currently, there is no trend towards a decrease in the content of CQAs or depletion of their composition. There was only one difference in the composition of CQAs: 3-CQA was not detected in Sr-L1 during 2014–2019, but was found in the samples of 2020–2022, although in small amounts. For the entire period of observation, the maximum content of the total CQAs in the calli of the Sr-L1 line was 40.61 mg/g dry weight, which was obtained in March 2022.

**Table 2:** Content of CQA derivatives (mg/g dry weight ± SE) in the callus line Sr-L1 and leaves of *S. radiate.* Results were obtained as an average of ten biological samples from different passages for callus line and three biological samples for leaves, with three analytical replicates each.

Peak Number	Metabolite	Abbreviation	Sr-L1	Leaves
**1**	3-*O*-caffeoylquinic acid	3-CQA	0.02 ± 0.01	0.31 ± 0.04
**2**	5-*O*-caffeoylquinic acid	5-CQA	7.54 ± 0.80	7.29 ± 0.06
**3**	4-*O*-caffeoylquinic acid	4-CQA	0.04 ± 0.00	0.19 ± 0.03
**4**	*cis*-5-*O*-caffeoylquinic acid	*cis*-5-CQA	0.03 ± 0.01	0.07 ± 0.01
**5**	5-*O*-*p*-coumaroylquinic acid		0.45 ± 0.03	0.26 ± 0.01
	*Total monoacyl derivatives*		*8.05* ± *0.79*	*8.11* ± *0.07*
**6**	3,4-*O*-dicaffeoylquinic acid	3,4-diCQA	0.29 ± 0.03	0.77 ± 0.05
**7**	3,5-*O*-dicaffeoylquinic acid	3,5-diCQA	18.52 ± 1.98	10.28 ± 0.37
**8**	*cis*-3,5-*O*-dicaffeoylquinic acid	*cis*-3,5-diCQA	0.10 ± 0.03	0.09 ± 0.01
**9**	4,5-*O*-dicaffeoylquinic acid	4,5-diCQA	0.40 ± 0.05	1.34 ± 0.12
**10**	3-*O*-*p*-coumaroyl-5-*O*-caffeoylquinic acid		0.24 ± 0.04	0.06 ± 0.01
**11**	3-*O*-caffeoyl-5-*O*-*p*-coumaroylquinic acid		0.34 ± 0.03	0.06 ± 0.01
	*Total diacyl derivatives*		*19.90* ± *2.03*	*12.60* ± *0.39*
	**Total**		**27.95** ± 1.86	**20.71** ± 0.31

### 2.4. Determination of Lignol Derivatives

The HPLC-UV-MS analysis of extracts from the Sr-L1 callus line of *S. radiata* showed the presence of three interesting peaks (**12–14**) (Figure 3), which were absent in leaf extracts. These components displayed absorption maxima at nearly 266 nm and were identified as monolignol (**12, 13**) and dilignol (**14**) derivatives on the basis of their detailed MS investigation (Table 3). The UV profile and MS data of component **13** corresponded to those described for syringin (sinapyl alcohol hexoside, also known as eleutheroside B) [34,35,36]. In particular, the perfectly shown negative [M+CH_3_COO]^−^ ions at *m/z* 431.1594 and positive [M+Na]^+^ ions at *m/z* 395.1329 of peak **13** were exactly matched to the molecular formula of syringin (C_17_H_24_O_9_). The negative MS^2^ spectrum of [M+CH_3_COO]^−^ precursor ions demonstrated two main signals at *m/z* 371 and 209, corresponding to the fragments produced by consistent elimination of acetic acid and dehydrated hexose moiety, with the compositions [M-H]^−^ and [M-H-C_6_H_10_O_5_]^−^, respectively (Table 3). In addition, the MS^3^ stage of ions at *m/z* 209 produced the fragmentation pattern that coincided with typical ion fragmentation for sinapyl alcohol [36]. Component **12** displayed the mass spectrometric behavior similar to that of component **13**, with a slight difference.

**Table 3:** List of lignol derivatives identified in the methanol extracts of Sr-L1 callus culture of *S. radiata* by their retention times, UV and MS data, and by comparison with published data.

Peak No. *	*t*_R_ (min)	λ_max_ (nm)	Detected Ions Composition	*m/z* Detected	*m/z* Calculated	Molecular Formula	MS^2^ Fragmentation (% Base Peak) (*m/z*)	MS^3^ Fragmentation (% Base Peak) (*m/z*)	Assignment
**12**	11.7	258	[M+CH_3_COO]^−^	401.1462	401.1453	C_16_H_22_O_8_	341(46), 221(2), **179(100)** **, 164(3), 161(3), 146(4)	164(62), 161(100), 146(43)	Coniferyl alcohol *O*-hexoside (coniferin, coniferoside, and abietin)
			[M+Na]^+^	365.1221	365.1207		-	-	
**13**	12.9	266	[M+CH_3_COO]^−^	431.1594	431.1573	C_17_H_24_O_9_	371(16), 221(8), **209(100)**, 194(9), 179(3), 176(4), 161(2)	194(100), 191(32), 179(3), 176(19), 161(3)	Sinapyl alcohol *O*-hexoside (syringin and eleutheroside B)
			[M+Na]^+^	395.1329	395.1313		-	-	
**14**	21.3	275	[M-H]^−^	579.2096	579.2083	C_28_H_36_O_13_	**417(100)**, 181(5)	402(40), 387(3), 371(3), 205(3), 181(100), 175(3), 166(38), 151(15)	Syringaresinol-*O*-hexoside (eleutheroside E1)
			[M+Na]^+^	603.2082	603.2054		-	-	

* The peaks are numbered as shown in Figure 3. ** Ions subjected to subsequent MS^n^ fragmentation are shown in bold.

The calculated molecular formula (using HRMS) of component **12** was C_16_H_22_O_8_, which had one less methoxy group compared to compound **13**, defined as syringin. We designated the component **12** as coniferyl alcohol *O*-hexoside, also known as coniferin [37]. The MS^2^ fragmentation pattern of compound **12** was also consistent with previously published data for coniferin [37].

Similarly, component **14** was identified as syringaresinol-*O*-hexoside (also known as eleutheroside E1), the UV and MS characteristics of which were described previously [38,39]. In the ESI conditions, component **14** showed the formation of deprotonated ions [M-H]^−^ at *m/z* 579.2096 and positive sodium adduct ions [M+Na]^+^ at *m/z* 603.2082. These data are in good agreement with the molecular formula of syringaresinol-hexoside (C_28_H_36_O_13_). MS^2^ fragmentation of [M-H]^−^ showed an intensive signal at *m/z* 417 due to the loss of the hexoside moiety, and the MS^3^ stage of precursor ions at *m/z* 417 provided fragmentation patterns identical to syringaresinol [34,40].

### 2.5. Determination of Flavonoid and Dihydrostilbene Derivatives

Peaks **15–29** were detected only in leaves of *S. radiate* and were absent in callus extracts. A DAD profile of leaf extracts at 325 nm in conjunction with extracted ion chromatograms (Figure 2B and Figure 3B) indicated several peaks corresponding to flavonoid glycosides (flavones **15–20**, **22**, **26** and **27**; flavonoles **23** and **24**) (Table 4). The complete structural assignments of these peaks were performed by means of comparison of all collected UV and MS data with previously published information on the genus *Scorzonera* [26,40,41].

**Table 4:** List of flavonoid and dihydrostilbene derivatives identified in the methanol extracts of *S. radiata* leaves by their retention times, UV and MS data, and by comparison with published data.

Peak No. *	*t*_R_ (min)	λ_max_ (nm)	Detected Ions Composition	*m/z* Detected	*m/z* Calculated	Molecular Formula	MS^2^ Fragmentation (% Base Peak) (*m/z*)	Assignment
**15**	16.4	271, 338	[M-H]^−^	563.1429	563.1406	C_26_H_28_O_14_	503(11), 473(68), 443(98), 383(86), 353(100)	Apigenin-*C*-hexoside-*C*-pentoside I
			[M+H]^+^	565.1531	565.1552		-	
**16**	16.9	271, 238	[M-H]^−^	563.1430	563.1406	C_26_H_28_O_14_	503(5), 473(50), 443(95), 383(57), 353(100)	Apigenin-*C*-hexoside-*C*-pentoside II
			[M+H]^+^	565.1542	565.1552		-	
**17**	17.0	257sh., 269, 348	[M-H]^−^	447.0953	447.0933	C_21_H_20_O_11_	429(25), 357(82), 327(100)	Luteolin-6-*C*-glucoside (isoorientin)
			[M+H]^+^	449.1075	449.1079		-	
**18**	17.5	256sh., 268, 350	[M-H]^−^	447.0942	447.0933	C_21_H_20_O_11_	357(50), 327(100)	Luteolin-8-*C*-glucoside (Orientin)
			[M+H]^+^	449.1093	449.1079		-	
**19**	18.3	270, 339	[M-H]^−^	533.1325	533.1300	C_25_H_26_O_13_	515(15), 473(71), 443(100), 383(60), 353(56)	Apigenin-di-*C*-pentoside I
			[M+H]^+^	535.1427	535.1446		-	
**20**	18.7	271, 338	[M-H]^−^	533.1323	533.1300	C_25_H_26_O_13_	515(19), 473(85), 443(100), 413(16), 383(72), 353(68)	Apigenin-di-*C*-pentoside II
			[M+H]^+^	535.1463	535.1446		-	
**21**	18.9	285	[M-H]^−^	433.1523	433.1504	C_22_H_26_O_9_	271(100), 165(4)	Scorzodihydrostilbene C
			[M-H-C_6_H_10_O_5_]^−^	271.0968	271.0970		229(4), 165(100), 149(22)	
			[M+Na]^+^	457.1458	457.1475		-	
**22**	19.0	271, 337	[M-H]^−^	431.0998	431.0984	C_21_H_20_O_10_	341(6), 311(100)	Apigenin-8-*C*-glucoside (vitexin)
			[M+H]^+^	433.1119	433.1129		-	
**23**	19.2	255, 265sh., 360	[M-H]^−^	463.0898	463.0882	C_21_H_20_O_12_	301(100), 271(2), 179(3), 151(1)	Quercetin-3-*O*-galactoside (hyperoside)
			[M+H]^+^	465.1011	465.1028		-	
**24**	19.5	255, 265sh., 358	[M-H]^−^	463.0901	463.0882	C_21_H_20_O_12_	301(100), 271(1), 179(3), 151(2)	Quercetin-3-*O*-glucoside (isoquercitrin)
			[M+H]^+^	465.1015	465.1028		-	
**25**	19.9	284	[M-H]^−^	463.1626	463.1610	C_23_H_28_O_10_	301(100), 165(2)	Scorzodihydrostilbene A
			[M-H-C_6_H_10_O_5_]^−^	301.1072	301.1081		283(10), 259(4), 165(100), 149(48)	
			[M+Na]^+^	487.1571	487.4580		-	
**26**	20.0	Nd**	[M-H]^−^	431.1005	431.0984	C_21_H_20_O_10_	341(2), 311(100)	Apigenin-6-*C*-glucoside (isovitexin)
			[M+H]^+^	433.1141	433.1129		-	
**27**	22.2	269, 340	[M-H]^−^	401.0891	401.0878	C_20_H_18_O_9_	341(29), 311(100)	Apigenin-*C*-pentoside
			[M+H]^+^	403.1037	403.1024		-	
**28**	22.8	280	[M-H]^−^	477.1777	477.1766	C_24_H_30_O_10_	315(100)	Scorzodihydrostilbene D
			[M+Na]^+^	501.1755	501.1737		-	
**29**	23.7	285	[M-H]^−^	477.1785	477.1766	C_24_H_30_O_10_	357(23), 315(100), 163(1)	Scorzodihydrostilbene B
			[M-H-C_6_H_10_O_5_]^−^	315.1229	305.1238		299(22), 297(44), 281(25), 163(100), 149(31)	
			[M+Na]^+^	501.1751	501.1737		-	

* The peaks are numbered as shown in Figure 2 and Figure 3. ** Not detected.

Compounds related to peaks **15** and **16** were identified as a pair of isomers by the identity of their UV and MS data (Table 4). The molecular formula of **15** and **16** was calculated as C_26_H_28_O_14_. The MS^2^ spectra of [M-H]^−^ at *m/z* 563 exhibited the pattern of fragmentation, which is typical for di-*C*-glycoside conjugates: [(M-H)-60]^−^ at *m/z* 503, [(M-H)-90]^−^ at *m/z* 473, [(M-H)-120]^−^ at *m/z* 443, [Agl+113]^−^ at *m/z* 383, and [Agl+83]^−^ at *m/z* 353. The presence of [(M-H)-60]^−^ fragments in the MS^2^ spectra corresponded to elimination of the *C*-pentosyl ring moiety, whereas fragment ions with the composition [(M-H)-90]^−^ and [(M-H)-120]^−^ indicated the *C*-hexosylated molecule.

Thus, compounds **15** and **16** were identified as isomers of apigenin-*C*-hexoside-*C*-pentoside, namely isoschaftoside and schaftoside, respectively, found earlier in the Asteraceae family [40,42]. The remaining *C*-glycosides were determined the same way using a well-known algorithm [43,44]. On the basis of retention times and UV and MS data (Table 4), these compounds were identified as luteolin-6-*C*-glucoside (isoorientin) (**17**), luteolin-8-*C*-glucoside (orientin) (**18**) [26,40,41], two isomers of apigenin-di-*C*-pentoside (**19** and **20**), apigenin-8-*C*-glucoside (vitexin) (**22)**, apigenin-6-*C*-glucoside (isovitexin) (**26**) [41,42], and apigenin-*C*-pentoside (**27**). Components corresponding to peaks **23** and **24** were identified as two isomers of flavonol *O*-glycoside due to typical UV profiles and MS^2^ fragmentations of deprotonated ions (Table 4). As a result, quercetin-3-*O*-galactoside (hyperoside) (**23**) and quercetin-3-*O*-glucoside (isoquercitrin) (**24**) were identified, according to [40,41,42]. Flavonoid derivative content in extracts of *S. radiata* leaves is presented in Table 5.

A DAD profile of leaf extracts at 280 nm demonstrated four low-intensity peaks matched to dihydrostilbene glycosides. The compound corresponding to peak **21** has the molecular formula C_22_H_26_O_9_, as determined by accurate mass measurement of its deprotonated molecules [M-H]^−^ at *m/z* 433.1523 and sodium adduct ions [M+Na]^+^ at *m/z* 457.1458 (Table 3, Figure 3B). MS^2^ investigation of [M-H]^−^ at *m/z* 433 showed major fragment ions formed by the elimination of the *O*-hexoside residue [M-H-C_6_H_10_O_5_]^−^. Based on these data, compound **21** was identified as scorzodihydrostilbene C, first discovered in *S. radiata* and described by Wang et al. [5]. Using the same method, components **25**, **28**, and **29**, with similar UV and MS^2^ profiles, were assigned as scorzodihydrostilbene A, scorzodihydrostilbene D, and scorzodihydrostilbene B, respectively [5].

## 3. Discussion

Efficient production of secondary metabolites requires their stable production by plant cell cultures during long-term cultivation. In this regard, the study of the biosynthetic properties stability of cultured cells should be carried out during long-term cultivation ([45]—Section “Increasing cell productivity: what next?”). Extremely high yields of secondary metabolites are desirable, but they are difficult to attain in combination with such characteristics as long-term stability and vigorous growth [45,46]. There are few examples of long-term preservation of high productivity of secondary metabolites in plant cell cultures. The most prominent examples are isoflavones (25 years of stable production, [47]), naphthoquinones and rosmarinic acid derivatives (15 years, [45]), and anthraquinones (14 years, [48]). The callus line Sr-L1 was established 21 years ago. From 2014 to the present, it has been under intense scrutiny for the production of secondary metabolites. The composition of CQAs in the Sr-L1 culture was identical to that in the *S. radiata* leaves. The total content of CQA derivatives in Sr-L1 was 1.35 times higher than in leaves (27.95 vs. 20.71 mg/g dry weight). Among CQAs, the highest content was detected for 5-CQA (7.54 mg/g dry weight) and 3,5-diCQA (18.52 mg/g dry weight) (Table 2).

These values are close to the maximum yields of CQAs previously obtained in cultured cells or tissues of various plant species. For example, 5-CQA was *produced in* the cell suspension culture of *Nicotiana plumbaginifolia* at the level of 4–6.5 mg/g dry weight; the *Fabiana imbricate* callus culture *contained* 5-CQA at the level of *4.6* mg/g dry weight, and the highest content (9.5 mg/g dry weight) was found in *Cichorium intybus* hairy roots, transformed with the pRi-1855 *Agrobacterium rhizogenes* strain (reviewed by Bulgakov et al. [46]). Likewise, the content of 3,5-diCQA was 3 mg/g dry weight in *Rhaponticum carthamoides hairy roots*; an in vitro culture of *Tanacetum vulgare* roots produced 10.2 mg/g dry weight of 3,5-diCQA [49]. Most likely, the maximum yield of 3,5-diCQA was achieved in the hairy root culture of *Cichorium intybus*, which amounted to 55.7 mg/g dry weight [46]. Interestingly, artichoke (the cultivated cardoon or *Cynara cardunculus* var. *altilis* DC (Asteraceae)) calli transformed in our laboratory with the *rolC* gene (which is a known inducer of secondary metabolism) produced predominantly 1,5-diCQA, 3,4-diCQA, and chlorogenic acid, but not 3,5-diCQA [33]. The total content of CQAs was 15.4 mg/g dry weight, which is less than that of the Sr-L1 line.

Regarding biological activity of the most abundant compound, 3,5-diCQA, it should be noted that considerable attention has been paid to this compound in recent years. Raineri et al. [50] reported that 3,5-diCQA treatment increased the level of the transcription factor Nrf2 (nuclear factor erythroid 2-related factor 2) that induces the expression of the heme oxygenase 1 (HO-1) with a subsequent inhibition of both C/EBP-α (late adipogenic transcription factor C/EBP) expression and adipocyte terminal differentiation. This molecular mechanism is probably common to other compounds containing a caffeic acid moiety [50], however, 3,5-diCQA seems to be more effective that other CQA derivatives [51]. Another important action of 3,5-diCQA is its beneficial effect in neurodegenerative diseases. In particular, 3,5-diCQA improved learning and alleviated memory deficits in mice by preventing neuronal apoptosis through the protection of mitochondrial activities and the repression of apoptotic signaling molecules [52]. 3,5-DCQA attenuates inflammation-mediated hypersensitivity to pain through inhibition of MCP3-induced JAK2/STAT3 (a protein module that induces the expression of genes regulating inflammation) signaling [53]. Therefore, 3,5-DCQA could be a potential therapeutic agent for alleviating inflammatory pain. Pang et al. [54] showed that the methyl derivatives of 5-CQA (MCGA) and 3,5-diCQA are potent antiproliferation agents in hepatocellular carcinoma cells, exceeding this effect of 5-CQA. MCGA and 3,5-diCQA perturb the expression of the HIF-1α-GLUT1/3-glycolysis pathway, thus inhibiting hepatocellular carcinoma proliferation and metastasis. The authors indicated these effects as a new research hotspot in carcinoma treatment.

It is also interesting to compare the chemical composition of the plants used in our experiments with the literature data. Previously, the chemical composition of *S. radiata* metabolites from Mongolia was studied by Wang et al. [5,26] and Tsevegsuren et al. [27]. Totally, these authors isolated 20 metabolites from the aerial parts of the plant, including 6 flavonoids, 5 dihydrostilbenes, and 9 derivatives of CQA. Overall, we identified 26 polyphenolic compounds in leaves of *S. radiata*, with the most significant differences between samples observed in the composition of flavonoids. Four of the five dihydrostilbenes first discovered by Wang et al. [5] in *S. radiata* from Mongolia, scorzodihydrostilbene A, B, C, and D, were also detected in Sakhalin plants. At the same time, only one flavonoid, luteolin-6-*C*-glucoside I (isoorientin), was detected in both samples, while five other flavonoids (scorzonerin A, scorzonerin B, violanthin, kaempferol 3-*O*-rutinoside, and rutin), previously identified in Mongolian plants [26,27], were not detected in Sakhalin samples. Ten flavonoids were found in Sakhalin samples, which are not described for Mongolian plants, and thus, they are described for the first time for *S. radiata* (Table 4 and Table 5). As shown in a recent review by Lendzion et al. [8], the aerial parts of various species of the *Scorzonera* genus contain a wide variety of flavonoids. Apigenin, luteolin, and quercetin, as well as apigenin-*C*-glycosides, luteolin-*C*-glycosides, luteolin-*O*-glycosides, and quercetin-*O*-glycosides are the most frequently found in *Scorzonera* species. Luteolin 6-*C*-glucoside (isoorientin), found in the present work and by Wang et al. [26] in *S. radiata* plants, was also identified in nine other *Scorzonera* species [8].

As for CQAs, their composition also differs in the samples from Mongolia and Sakhalin Island. Quinic acid and eight CQA derivatives, two monoacyl and six diacyl ones, were isolated from Mongolian samples of *S. radiata* [26,27]. We identified 11 CQAs in *S. radiata* originating from Sakhalin, namely 5 monoacyl and 6 diacyl derivatives (Table 1, Figure 4). Four of them, 5-CQA, 5-*O*-p-coumaroylquinic acid, 3,5-diCQA, and 4,5-diCQA, were common for both Mongolian and Sakhalin samples. CQAs easily undergo structural transformation due to the spontaneous migration of caffeoyl residues among the hydroxyl groups of quinic acid under heating [16,55,56,57,58]. Taking into account that CQA derivatives are unstable under illumination and heating, the mildest methods of chemical analysis were used in the present investigation. Thus, 3,4-diCQA and monoesters of quinic acid, 3-CQA and 4-CQA, were identified for *S. radiata* plants for the first time (Table 1). Similarly, mixed diesters such as 3-*O*-*p*-coumaroyl-5-*O*-CQA and 3-*O*-caffeoyl-5-*O*-*p*-coumaroylquinic acid were identified in *S. radiata* for the first time (Table 1).

## 4. Materials and Methods

### 4.1. Plant Material and Callus Culture

Samples of *Scorzonera radiata* Fisch. (Asteraceae) were collected on Sakhalin Island in 2001 during a joint Russian–American–Japanese expedition (International Sakhalin Island Project, https://www.burkemuseum.org/static/okhotskia/isip/Results/reports/nsf/02report.htm: accessed on 17 October 2022). The plants were identified at the Department of Botany of the Federal Scientific Center of the East Asia Terrestrial Biodiversity (Vladivostok, Russia). Callus cultures were established from young leaves of the collected plants in the laboratory of the research vessel Akademik Oparin. Calli were established as described previously [59]. Callus cultures were cultivated in 100 mL Erlenmeyer flasks on W_B/A_ medium at 25 °C in the dark at 30-day subculture intervals. The W_B/A_ medium contained standard Murashige and Skoog macrosalts, microsalts, and Fe-EDTA, with the exception of NH_4_NO_3_, the concentration of which was decreased up to 400 mg/L. The following components were added to the W_B/A_ medium (mg/L): thiamine-HCl (0.2), nicotinic acid (0.5), pyridoxine-HCl (0.5), meso-inositol (100), peptone (100), sucrose (25,000), agar (6000), 6-benzylaminopurine (0.5), and α-naphthaleneacetic acid (2.0). All reagents were obtained from Sigma-Aldrich (St. Louis, MO, USA, “Tissue Culture Grade”). The actively growing callus culture of leaf origin was selected and designated as the Sr-L1 line. The growth index of Sr-L1 was calculated as W_f_-W_i_/W_i_, where W_f_ is the final biomass and W_i_ represents the inoculum biomass (0.2 g). The obtained callus culture is maintained by subculturing in the Collection of Plant Cell Cultures at the Federal Scientific Center of the East Asia Terrestrial Biodiversity.

### 4.2. Chemicals

All used solvents were of high-performance liquid chromatography (HPLC) grade. Chemical standards for the quantitative determination of CQAs (chlorogenic acid, CAS number: 327-97-9, and cynarin, 30964-13-7) and flavonoids (rutin trihydrate, CAS number: 250249-75-3) were purchased from Sigma-Aldrich (St. Louis, MO, USA).

### 4.3. Sample Preparation for Analytical Chromatography

*Scorzonera radiata* plants were brought from Sakhalin Island and transplanted into pots. Fresh leaves were collected for analysis, washed in distilled water, and subjected to convection drying at 50 °C in the darkness to a constant weight. Callus tissue was collected from the culture vessels and immediately dried under the same conditions. Leaf and callus samples were prepared for further analysis in the same way. Dried and powdered samples (about 100 mg, in duplicate) were sonicated at room temperature for 20 min in 3 mL of methanol, equilibrated for 3 h in the darkness, and then centrifuged (15,000× *g*, 10 min). The supernatant was filtered, and the residue was re-extracted once more in the same manner. The extracts were combined, cleared with a 0.45 μm membrane (Millipore, Bedford, MA, USA), and used for HPLC analysis. Then, 1 mL aliquots were employed for UV irradiation.

### 4.4. UV Irradiation

UV irradiation was performed to confirm the presence of cis isomers, since chlorogenic acid derivatives are known to undergo trans–cis isomerization under UV light [13]. If, after UV irradiation, the compound increases the peak intensity on the HPLC chromatogram, this means that this compound is indeed a cis isomer. The prepared samples of plant material (1 mL of each) were irradiated under a shortwave UV lamp at 245 nm for 40 min and used for HPLC analysis.

### 4.5. Analytical Chromatography and Mass Spectrometry

HPLC analysis of the samples was performed at the Instrumentation Center for Biotechnology and Genetic Engineering of the Federal Scientific Center for Terrestrial Biodiversity of East Asia using a 1260 Infinity analytical HPLC system (Agilent Technologies, Santa Clara, CA, USA) equipped with a G1315D photodiode array detector, G1311C quaternary pump, G1316A column oven, and G1329B auto sampler. The HPLC system was interfaced with an ion trap mass spectrometer (Bruker HCT ultra PTM Discovery System, Bruker Daltonik GmbH, Bremen, Germany) equipped with an electrospray ionization (ESI) source. The MS analyses were carried out with negative ions detection. The following settings were used: the range of *m/z* detection was 100–1000 and the drying gas (N_2_) flow rate was 8.0 L/min. The nebulizer gas (N_2_) pressure was 175 kPa, the ion source potential was −4.0 kV, and the drying gas temperature was 325 °C. Tandem mass spectra were acquired in Auto-MS^n^ mode (smart fragmentation) using a ramping of the collision energy. The fragmentation amplitude was set to 1 V. If necessary, multistage MS experiments were performed for the parent ions of the monitoring compounds.

HPLC with high-resolution mass spectrometry (HPLC-UV-HRMS) was carried out using a LCMS-IT-TOF tandem mass spectrometer (Shimadzu, Japan) including LC-20AD Prominence liquid chromatograph (Shimadzu, Japan) and an ion trap/time-of-flight mass spectrometer. The mass spectra were recorded applying electrospray ionization (ESI) with simultaneous negative and positive ion detection with a resolution of 12,000. The following settings were used: the range of *m/z* detection was 100–1000, the drying gas (N_2_) pressure was 195 kPa, the nebulizer gas flow rate was 1.5 L/min, the ion source potential changed from −3.8 to 4.5 kV, and the interface temperature was 200 °C.

An analytical Zorbax C18 column (150 mm, 2.1 mm i.d., 3.5 μm part size, Agilent Technologies, Santa Clara, CA, USA) was applied for separation. The column temperature was maintained at 40 °C. The mobile phase consisted of a gradient elution of 0.1% aqueous acetic acid (A) and acetonitrile (B). The gradient profile with a flow rate of 0.2 mL/min was the following: 0 min 5% B; 20 min 30% B; 30 min 100% B; and then eluent B until 40 min. The injection volume was 1–5 μL. UV spectra were recorded with a DAD detector in the range between 200 and 400 nm. Chromatograms for quantification were acquired at a wavelength of 325 nm.

All identified phenolic acids and flavonoid glycosides were quantified using HPLC with UV detection at a wavelength of 325 nm on the basis of four-point regression curves built with the reference commercial standards. For quantification of CQAs, two external standards were used: chlorogenic acid for monoacyl derivatives and cynarin for diacyl derivatives. Quantitative data of all flavonoid glycosides were obtained using rutin trihydrate solution as the external standard. The amount of each individual compound was calculated to correct for the difference in molecular weights.

### 4.6. Statistical Analysis

All values were expressed as the mean ± SE using Statistica 10.0 (StatSoft Inc., Tulsa, OK, USA).

## 5. Conclusions

The Sr-L1 callus line produced CQAs and lignol derivatives but did not produce flavonoids and dihydrostilbenes. The leaves of *S. radiata*, in turn, did not contain lignols. The high content of CQAs and the exceptional stability of biosynthesis make the callus culture of *S. radiata* Sr-L1 a promising source for the development of drugs and nutraceuticals for daily and dietary nutrition. This is especially important in connection with the search for effective means to combat obesity and other metabolic disorders, as well as potential activity of 3,5-diCQA in improving cognitive functions. From a biotechnological point of view, it is interesting to note that the Sr-L1 line produces 3,5-diCQA in high amounts, 1.9% of the dry cell weight, for many years. Apparently, this observation will contribute to the increased interest of biotechnology companies in this class of secondary metabolites.

## Figures and Tables

**Table 5:** Content of flavonoid derivatives (mg/g dry weight) in leaves of *S. radiata*. The results are presented as mean ± SE of three biological samples.

Peak Number	Metabolite	Content
**15**	Apigenin-*C*-arabinoside-*C*-glucoside I	0.61 ± 0.05
**16**	Apigenin-*C*-arabinoside-*C*-glucoside II	0.99 ± 0.07
**17**	Luteolin-6-*C*-glucoside I	1.32 ± 0.05
**18**	Luteolin-8-*C*-glucoside II	0.57 ± 0.09
**19**	Apigenin-di-*C*-arabinoside I	0.31 ± 0.03
**20**	Apigenin-di-*C*-arabinoside II	2.02 ± 0.19
**22**	Apigenin-*C*-glucoside I	0.79 ± 0.10
**23**	Quercetin-*O*-glucoside I	0.25 ± 0.02
**24**	Quercetin-*O*-glucoside II	0.15 ± 0.01
**26**	Apigenin-*C*-glucoside II	0.31 ± 0.02
**27**	Apigenin-*C*-arabinoside	0.67 ± 0.04
	**Total**	**7.98** ± **0.29**
